# BRCA-1 and BRCA-2 mutation bedside detection and breast cancer clinical primary prevention

**DOI:** 10.3389/fgene.2013.00039

**Published:** 2013-03-26

**Authors:** Sergio Stagnaro, Simone Caramel

**Affiliations:** Department of Advanced Research, International Society of Quantum Biophysical Semeiotics, Board of directors, Research CenterLancenigo, Italy

As regards the interesting discovery of a novel BRCA1 mutation in a family of Palestinian Arabian origin (Kadouri et al., 2007), we would like to state that all gene mutations bring about necessarily local biological activity modification, otherwise, gene mutations would be meaningless innocent bystanders (Stagnaro-Neri and Stagnaro, 1995; Kadouri et al., 2007; Stagnaro, 2008).

In the present article we suggest an original clinical tool for the diagnosis of Inherited Real Risk (IRR) of breast cancer which can support the current sophisticated ways for breast cancer risk assessment such as, e.g., the traditional breast physical examination, and the denaturing high performance liquid chromatography (DHPLC) to screen for mutations of BRCA1- BRCA-2 (Kadouri et al., 2007), which have been linked to hereditary breast and ovarian cancer, and inheriting this mutation increases the risk of developing breast/ovarian cancer. Furthermore, this last evaluation is expensive for National Health Service (NHS), and not applicable for all women and men.

Quantum Biophysical Semeiotics (QBS) theory provides a clinical, reliable method both for bed-side diagnosis and breast cancer primary and pre-primary prevention, according to the Manuel's Story (http://www.sisbq.org/qbs-magazine.html) (Stagnaro and Stagnaro-Neri, 2004a).

QBS is a new discipline in medical field and an extension of the classical medical semeiotics with the support of quantum and complexity theories (Caramel and Stagnaro, 2011). It is a scientific trans-disciplinary approach that is based on the “Congenital Acidosic Enzyme-Metabolic Histangiopathy” (CAEMH) (Stagnaro and Caramel, 2010), a unique mitochondrial cytopathy that is present at birth and subject to medical therapy. The presence of intense CAEMH in a well-defined area (e.g., myocardium) is due to gene mutations in both n-DNA and mit-DNA.

This is the basis for one or more QBS constitutions (Stagnaro and Stagnaro-Neri, 2004b), in our case, Oncological Terrain (Stagnaro, 2004), which could bring about their respective IRR, i.e., IRR of cancer (Stagnaro, 2009; Stagnaro and Caramel, 2012, 2013a,b). The QBS method allows the clinical and pre-clinical diagnosis of the most severe diseases such as the IRR of breast cancer (Stagnaro, 2004, 2005a,b,c,d), which is achieved in the easier way through the auscultatory percussion of the stomach (Stagnaro, 1985, 2005e). The patho-physiology of QBS reflexes is based upon local microvascular conditions. In case of genetic alteration of both DNAs, intense CAEMH, and IRR of breast cancer there is a microcirculatory remodeling, worsened by well-known environmental risk factors, due to vasomotility and vasomotion impairment (e.g., functional imperfection) and structural obstructions, i.e., pathological Endoarteriolar Blocking Devices (EBDs) and Arteriovenous Anastomosis (AVA) (Stagnaro and Stagnaro-Neri, 2004a; Stagnaro, 2009; Stagnaro and Caramel, 2010).

With the aid of QBS method, physicians can bedside recognize, in an easy, quick, and reliable manner, the possible presence of maternally-inherited Oncological Terrain, and Oncological terrain-dependent, IRR, based on the presence of typical microcirculatory remodeling of mamma microvessels, due to newborn-pathological, type I, subtype (a) Oncological, EBDs (Stagnaro and Stagnaro-Neri, 2004a; Stagnaro and Caramel, 2010), conditio sine qua non of breast cancer (Stagnaro, 2009).

In spite of genetic testing, bedside ascertaining particularly breast cancer IRR in well-defined breast quadrant(s) allows physicians to perform an efficient malignancy primary prevention in a few minutes. In addition, testing for mutations breast cancer susceptibility genes or for their diminished expression adds to the ability to assess breast cancer IRR at an individual level, because local biological activity, examined with the aid of QBS, results abnormal.

Really, by means of sophisticated semeiotics images we cannot localize in mamma quadrant(s) the possible IRR of breast cancer, in BRCA 1, or BRCA1 mutation, E1373X in exon 12 and BRCA 2 in exons 9, 10, 11, 17, 18, and 23 positive women (and men) (Stagnaro-Neri and Stagnaro, 1995; Stagnaro, 2004, 2009; Stagnaro and Stagnaro-Neri, 2004a,b; Stagnaro, 2008; Stagnaro and Caramel, 2010; Caramel and Stagnaro, 2011). In turn, by means of QBS method, physicians can clinically recognize firstly the Oncological Terrain in a quantitative way (Stagnaro, 2011a), and then, but not in all cases, the IRR of breast cancer: individuals with Oncological Terrain do not show necessarily also breast cancer IRR (Stagnaro, 2005a,b,c,d).

As a matter of fact, breast cancer, bedside subdivided regarding ERs and cytokine content, involves exclusively the subject positive for Oncological Terrain (Stagnaro, 2004; Stagnaro and Stagnaro-Neri, 2004a,b; Stagnaro and Caramel, 2010; Caramel and Stagnaro, 2011). We know that multiple cytokines, e.g., Interleukin 12 (IL-12), and Interleukin 23 (IL-23) were over-expressed in ER-negative breast carcinoma and that the three major cytokines—MCP-1, MIP-1beta and IL-8—were correlated to inflammatory cell component, which could account for the aggressiveness of these tumors (Stagnaro-Neri and Stagnaro, 1995; Stagnaro and Stagnaro-Neri, 2004a; Stagnaro, 2005a,b,c,d, 2008; Stagnaro and Caramel, 2010; Caramel and Stagnaro, 2011). An early bedside diagnosis of breast cancer IRR allows both a pre-primary and primary prevention (Stagnaro and Caramel, 2013c) and detection, corroborated by several other QBS signs such as the bedside evaluation of glycocalyx (Stagnaro, 2011b).

Interestingly, from clinical and experimental data there is an emerging evidence that familial breast cancers, including BRCA1 and its related forms, could be estrogen-sensitive and interactions between BRCA1 gene expression and estrogens have been reported (Zheng et al., 2001; Lindgren et al., 2002; Venkitaraman, 2002). Moreover, BRCA1 blocked the expression of two endogenous estrogen-regulated gene products in human breast cancer cells (Ma et al., 2005). These knowledge accounts for the reason QBS allows differential diagnose between positive and negative breast cancer at the bedside.

In addition, the presence of breast ER, even localized in a mamma quadrant, is bedside recognized rapidly and in a reliable manner, by occurrence of type I, associated, microcirculatory activation, subsequent to oestrogene secretion pick test, i.e., digital pressure upon Estrogen-RH centers, lasting 15 s (Stagnaro and Stagnaro-Neri, 2004a).

On the contrary, in absence of ER_s_ local microcirculatory blood-flow persists unchanged, evaluated as the latency time of mamma-gastric aspecific reflex (Stagnaro and Stagnaro-Neri, 2004a; Stagnaro, 2005a,b,c,d), As far as assessing cytokine levels in the breast (or in all other biological systems), it is sufficient to know that, in health, intense breast trigger-points stimulation by finger nail, i.e., it brings about a gastric aspecific reflex after 10 s latency time. On the contrary, in presence of cytokines, latency time of this reflex is lower and it results inversely related to the underlying cytokine level.

Mutations of BRAC1 and BRAC2 genes have been linked to hereditary breast and ovarian cancer. Inheriting these mutations, the risk of developing breast/ovarian cancer generally increases, but genetic tests cannot occur in every laboratory. QBS allows physicians to bedside recognize in quantitative way and precisely localize from birth both breast cancer IRR and the presence of BRCA-1 as well as BRCA-2 mutations. In conclusion, with the simple use of the stethoscope, QBS diagnostic method is useful for a large scale clinical diagnosis of Oncological Terrain-Dependent and breast cancer IRR, so allowing an effective primary and pre-primary prevention.

## References

[B1] Caramel S. Stagnaro S. (2011). Quantum biophysical semeiotics and mit-Genome's fractal dimension. J. Quantum Biophys. Semeiot. 1, 1–17

[B2] Kadouri L. Bercovich D. Elimelech A. Lerer I. Sagi M. Glusman G. (2007). A novel BRCA-1 mutation in Arab kindred from east Jerusalem with breast and ovarian cancer. BMC Cancer 7:14 10.1186/1471-2407-7-14 17233897 PMC1784098

[B3] Lindgren P. R. Backstrom T. Cajander S. Damber M. G. Mahlck C. G. SZhu D. (2002). The pattern of estradiol and progesterone differs in serum and tissue of benign and malignant ovarian tumours. Int. J. Oncol. 21, 583–589 12168103

[B4] Ma Y. M. Tomita Y. Fan S. Wu K. Tong Y. Zhao Z. (2005). Structural determinants of the BRCA1: estrogen receptor interaction. Oncogene 24, 1831–1846 10.1038/sj.onc.1208190 15674350

[B5] Stagnaro S. (1985). Auscultatory percussion of the cerebral tumour: diagnostic importance of the evoked potentials. Biol. Med. 7, 171–175

[B6] Stagnaro S. (2004). Genes and cancer: a clinical view-point. The Oncological Terrain. BMC Inform. Available online at: http://www.biomedcentral.com/1471-2105/5/21/comments#10454

[B8] Stagnaro S. (2005a). There is another clinical, and overlooked tool, reliable in breast cancer prognosis evaluation. Available online at: http://www.biomedcentral.com/1471-2407/5/70/comments#204473

[B9] Stagnaro S. (2005b). Bed-side evaluating breast cancer real risk. World J. Surg. Oncol. 3, 6716236180

[B10] Stagnaro S. (2005c). Mitochondrial bed-side evaluation: a new way in the war against cancer. Cancer Cell Int. Available online at: http://www.cancerci.com/content/5/1/34/comments

[B11] Stagnaro S. (2005d). A new way in the war against breast cancer, fortunately. Breast Cancer Res. Available online at: http://breast-cancer-research.com/content/7/2/R210/comments

[B12] Stagnaro S. (2005e). Clinical tool reliable in bedside early recognizing pancreas tumour, both benign and malignant. World J. Surg. Oncol. 3, 6216188022

[B13] Stagnaro S. (2008). Biological system functional modification parallels gene mutation. Available online at: http://blogs.nature.com/nm/spoonful/2008/03/gout_gene.html

[B14] Stagnaro S. (2009). Reale Rischio Semeiotico Biofisico. I Dispositivi Endoarteriolari di Blocco Neoformati, Patologici, Tipo I, Sottotipo a) Oncologico, e b) Aspecifico. Roma: Ediz. Travel Factory

[B15] Stagnaro S. (2011a). Rinaldi's sign in bedside diagnosing Di Bella's Oncological Terrain and overt cancer solid and liquid, in Proceedings of the 2nd Meeting of International Society of Quantum Biophysical Semeiotics (Siena), May 29–30.

[B16] Stagnaro S. (2011b). The role of glycocalyx in QBS diagnosis of Di Bella's Oncological Terrain. J. Quantum Biophys. Semeiot. Available online at: http://www.sisbq.org/uploads/5/6/8/7/5687930/oncological_glycocalyx2011.pdf

[B17] Stagnaro S. Caramel S. (2010). The role of mitochondria and mit-DNA in oncogenesis. Quantum Biosyst. 2, 250–281

[B23] Stagnaro S. Caramel S. (2012). Vascular calcification and Inherited Real Risk of lithiasis. Front. Endocrinol. 3:119 10.3389/fendo.2012.00119 23060859 PMC3464397

[B18] Stagnaro S. Caramel S. (2013a). Inherited Real Risk of Type 2 Diabetes Mellitus: bedside diagnosis, pathophysiology and primary prevention. Front. Endocrinol. 4:17 10.3389/fendo.2013.00017 23447747 PMC3581808

[B19] Stagnaro S. Caramel S. (2013b). The inherited real risk of coronary artery disease. Eur. J. Clin. Nutr. (in press). 10.1038/ejcn.2013.37 23612516

[B20] Stagnaro S. Caramel S. (2013c). The role of modified mediterranean diet and quantum therapy in oncological primary prevention. Curr. Nutr. Food Sci. 10.2174/1573401311309010011 PMC12878279 24402398

[B21] Stagnaro S. Stagnaro-Neri M. (2004a). Introduzione alla Semeiotica Biofisica. Il Terreno oncologico. Roma: Travel Factory S.R.L

[B22] Stagnaro S. Stagnaro-Neri M. (2004b). Le Costituzioni Semeiotico Biofisiche. Strumento clinico fondamentale per la prevenzione primaria e la definizione della Single Patient Based Medicine. Rome: Travel Factory

[B24] Stagnaro-Neri M. Stagnaro S. (1995). Cancro della mammella: prevenzione primaria e diagnosi precoce con la percussione ascoltata. Gazz. Med. It. – Arch. Sc. Med. 152, 447

[B25] Venkitaraman A. R. (2002). Cancer susceptibility and the functions of BRCA1 and BRCA2. Cell 108, 171–182 10.1016/S0092-8674(02)00615-3 11832208

[B26] Zheng L. Annab L. A. Afshari C. A. Lee W. H. Boyer T. G. (2001). BRCA1 mediates ligand-independent transcriptional repression of the estrogen receptor. Proc. Natl. Acad. Sci. U.S.A. 98, 9587–9592 10.1073/pnas.171174298 11493692 PMC55496

